# A Stochastic View of Spliceosome Assembly and Recycling in the Nucleus 

**DOI:** 10.1371/journal.pcbi.0030201

**Published:** 2007-10-26

**Authors:** José Rino, Teresa Carvalho, José Braga, Joana M. P Desterro, Reinhard Lührmann, Maria Carmo-Fonseca

**Affiliations:** 1 Instituto de Medicina Molecular, Universidade de Lisboa, Lisbon, Portugal; 2 Department of Cellular Biochemistry, Max-Planck-Institute of Biophysical Chemistry, Göttingen, Germany; National Cancer Institute, United States of America

## Abstract

How splicing factors are recruited to nascent transcripts in the nucleus in order to assemble spliceosomes on newly synthesised pre-mRNAs is unknown. To address this question, we compared the intranuclear trafficking kinetics of small nuclear ribonucleoprotein particles (snRNP) and non-snRNP proteins in the presence and absence of splicing activity. Photobleaching experiments clearly show that spliceosomal proteins move continuously throughout the entire nucleus independently of ongoing transcription or splicing. Using quantitative experimental data, a mathematical model was applied for spliceosome assembly and recycling in the nucleus. The model assumes that splicing proteins move by Brownian diffusion and interact stochastically with binding sites located at different subnuclear compartments. Inhibition of splicing, which reduces the number of pre-mRNA binding sites available for spliceosome assembly, was modeled as a decrease in the on-rate binding constant in the nucleoplasm. Simulation of microscopy experiments before and after splicing inhibition yielded results consistent with the experimental observations. Taken together, our data argue against the view that spliceosomal components are stored in nuclear speckles until a signal triggers their recruitment to nascent transcripts. Rather, the results suggest that splicing proteins are constantly diffusing throughout the entire nucleus and collide randomly and transiently with pre-mRNAs.

## Author Summary

Understanding the genomic program of an organism requires knowledge of how the information encoded in DNA is processed to generate messenger RNAs that can be translated into proteins. The initial products of gene transcription are extensively modified in the cell nucleus, and a major processing reaction consists of splicing of specific sequences from the middle of the primary transcripts. Splicing is catalyzed by the spliceosome, a large complex composed of five small RNAs and over 100 different proteins. Spliceosomes form anew on primary transcripts and disassemble after splicing, but what triggers the recruitment of individual spliceosomal components to selected gene products is unclear. Here, we have combined imaging and computational approaches to address this question. We obtained quantitative experimental data on the mobility and subnuclear distribution of splicing proteins before and after splicing inhibition, and we applied mathematical models to analyze and interpret the results. We conclude that spliceosomal components do not require a signal in order to be recruited to nascent transcripts. Our results favor the view that splicing proteins are constantly diffusing throughout the entire nucleus and collide randomly and transiently with primary gene products.

## Introduction

The spliceosome is the multi-megadalton machine that catalyses pre-mRNA splicing. The building blocks of the spliceosome are uridine-rich small nuclear RNAs (U snRNAs) packaged as ribonucleoprotein particles (snRNPs) that function in conjunction with numerous non-snRNP proteins [1,2]. The major spliceosomal small nuclear ribonucleoprotein particles are the U1, U2, U5, and U4/U6 snRNPs. Each snRNP consists of one or two U snRNAs (U1, U2, U5, and U4/U6 snRNAs) bound by a protein complex that comprises seven common Sm proteins and one or more proteins specific to each snRNP [3]. The Sm proteins B/B′, D1, D2, D3, E, F, and G are common to all spliceosomal snRNPs, except U6, and are arranged into a ring structure around a highly conserved single-stranded uridine-rich sequence of the snRNA [4–6]. The biogenesis of spliceosomal snRNPs involves a sequence of reactions that take place at different compartments within the cell. With the exception of U6, which acquires a γ-monomethyl phosphate cap and is restricted to the nucleus, the snRNAs are transcribed as initial precursors that are rapidly exported to the cytoplasm where they associate with Sm proteins [7]. Although in vitro Sm cores assemble readily on uridine-rich RNAs, in cells this process involves the survival of motor neurons (SMN) complex [8]. Assembly of the Sm core is a prerequisite for removal of the snRNA 3′ extension present in the precursor forms and hypermethylation of the 5′ m^7^ G cap to m^2,2,7^ G (m_3_G or TMG) [3,9]. The assembled Sm core and the modified cap then function as independent nuclear localization signals (NLS) for subsequent reimport into the nucleus. The m_3_G cap is recognized by Snurportin1, an import adaptor that interacts with importin-β [10,11], whereas the Sm core–mediated transport is linked to the nuclear import of SMN [12].

Spliceosomes form anew on nascent pre-mRNAs and disassemble after introns are excised and exons ligated. Thus, spliceosomal snRNPs and non-snRNP proteins in the nucleus can be either actively engaged in splicing or waiting for the next chance to assemble a spliceosome. When the mammalian cell nucleus is viewed with the electron microscope, spliceosomal components are detected in morphologically distinct structures termed Cajal bodies (CBs), interchromatin granule clusters (IGCs), and perichromatin fibrils [13].

The CB is highly enriched in snRNPs but is devoid of non-snRNP splicing proteins. Direct visualization of snRNPs in living cells shows that after import into the nucleus, the newly synthesized particles accumulate first in CBs and are later detected in the speckles [14]. Several lines of evidence indicate that maturation of newly synthesized snRNPs is completed in CBs [15]. Additionally, CBs are likely to be sites where snRNPs are recycled after spliceosome disassembly [16,17].

The perichromatin fibrils correspond to nascent transcripts and appear scattered throughout the nucleoplasm, excluding regions of condensed chromatin [18]. Perichromatin fibrils are often closely associated with the periphery of IGCs, making it impossible to distinguish the two structures within the speckled pattern that characterizes the distribution of splicing factors observed by fluorescence microscopy. Whereas snRNPs and splicing proteins detected on perichromatin fibrils most likely correspond to active spliceosomes, a large body of evidence indicates that the spliceosomal components localized in IGCs are not primarily involved in splicing [19]. Upon activation of a gene, the spliceosome rapidly assembles on the nascent pre-mRNA [20,21]. Conversely, gene inactivation increases the pool of “reserve” splicing factors that accumulate within enlarged IGCs. Consequently, the organization of the speckled pattern observed by fluorescence microscopy is a reflection of the transcriptional and splicing activity of the cell [21,22].

Although recruitment of splicing snRNPs and non-snRNP proteins to nascent transcripts has been visualized in several systems, how spliceosomal components are targeted to IGCs/nuclear speckles and what triggers their subsequent release and recruitment to nascent transcripts remain a controversial issue. Several lines of recent evidence suggest that the formation and maintenance of nuclear structures involved in transcription, splicing, DNA replication, and repair is governed by self-organization principles [23–26]. To determine whether the concept of self-organization applies to spliceosome assembly and recycling, we have compared the kinetics of snRNP and non-snRNP spliceosomal proteins as they move throughout the nucleus in the presence or absence of splicing activity. The quantitative experimental data obtained was then used for mathematical modeling. Our results are consistent with the view that splicing proteins move by Brownian diffusion and bind with specific on- and off-rates to other nuclear components.

## Results

### Kinetics of Splicing Proteins in the Living Cell Nucleus

**Figure 1:**
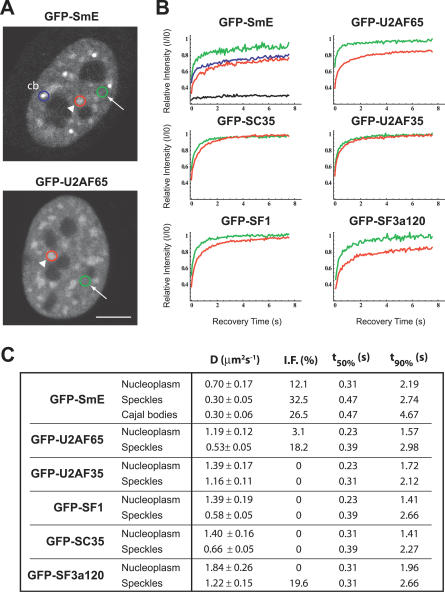
FRAP Analysis of Splicing Proteins in Different Subnuclear Compartments FRAP experiments were performed on HeLa cells expressing GFP-tagged splicing proteins, as indicated. (A) Images of representative cells. The circles illustrate bleach regions localized in the nucleoplasm (arrows, green circles), nuclear speckles (arrowheads, red circles), or CBs (cb, blue circle). Bar indicates 5 μm. (B) FRAP recovery curves of indicated GFP-tagged splicing proteins in the nucleoplasm (green curves), nuclear speckles (red curves), and CBs (blue curve). The fluorescence intensity *I* was monitored over time (*I* was corrected for the background intensity and the amount of total fluorescence lost during the bleach and imaging). Each recovery curve corresponds to a pool of three independent experiments, with ten different cells analyzed per experiment. The recovery for the immobile protein GFP-coilin-PABPN1 [60] is shown for comparison (black curve). (C) Experimental values obtained for the diffusion coefficient (*D*), apparent immobile fraction (*I.F.*), recovery time at 50% of initial fluorescence (*t_50%_*), and recovery time at 90% of initial fluorescence (*t_90%_*) in the speckles, CBs, and nucleoplasm.

In the nucleus of mammalian cells, components of the spliceosome are found distributed throughout the nucleoplasm, excluding nucleoli, and concentrated in nuclear speckles or IGCs (Figure 1A); additionally, splicing snRNPs are detected highly enriched in CBs [19,27,28]. To visualize trafficking of spliceosomal components between subnuclear compartments, the green fluorescent protein (GFP) was fused in frame to the amino terminus of a number of splicing proteins, including SmE, U2AF^65^, U2AF^35^, SF1, SC35, and SF3a120. Western blot analysis confirmed the expression of fusion proteins with the expected molecular weight, and fluorescence microscopy showed that the GFP tag did not affect the localization of the proteins.

Fluorescence recovery after photobleaching (FRAP) was used to analyze the relative mobility of the splicing proteins in the nucleoplasm, nuclear speckles, and CBs. Nuclear speckles are identified as structures of heterogeneous size and shape with higher fluorescence intensity than the nucleoplasm, whereas CBs appear as brighter spherical foci approximately 0.5 μm in diameter [19,27,28]. The fluorescence of a small area located in each of these subnuclear compartments was irreversibly photobleached using a high-intensity laser, and subsequent recovery due to movement of non-bleached molecules into the bleached area was recorded by time-lapse imaging. All GFP-tagged splicing proteins were found to be mobile in each of the subnuclear compartments, with half-time fluorescence recoveries under 0.5 s. For all proteins, the recovery time was systematically lower in the nucleoplasm than in the speckles (Figure 1B and 1C). Quantification of FRAP recovery curves yielded effective diffusion coefficient values ranging from 0.70 to 1.84 μm^2^ s^−1^ in the nucleoplasm and 0.30 to 1.22 μm^2^ s^−1^ in the nuclear speckles. Some of the proteins investigated (GFP-tagged SmE, U2AF^65^, and SF3a120) also showed significant immobile fractions in the speckles. In the case of GFP-U2AF^65^, the apparent immobile fraction recovered completely when FRAP analysis was extended to longer periods of time (Figure S1), indicating that the protein is transiently immobilized in the speckles.

**Figure 2:**
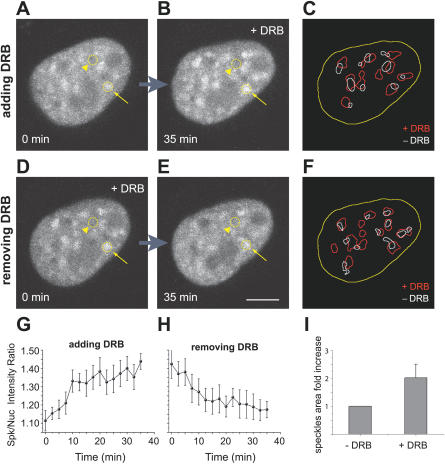
The Transcriptional Inhibitor DRB Induces a Reversible Accumulation of GFP-U2AF^65^ in Enlarged Nuclear Speckles Steady-state distribution of GFP-U2AF^65^ in the nucleus of a HeLa cell after addition (A,B) or removal (D,E) of DRB. (A,B) Depict the same cell imaged immediately after addition of DRB (A) and 35 min later (B). (D,E) Depict the same cell imaged immediately after removal of DRB (D) and 35 min later (E). The arrows point to a nuclear speckle, the arrowheads to the nucleoplasm. Bar indicates 5 μm. (G,H) Plot of the ratio between fluorescence intensities in the nuclear speckles (*n* = 6 speckles) and in the nucleoplasm over time after addition (G) or removal (H) of DRB. Error bars represent standard deviations. (C,F) Depict the threshold segmentations of images in (A,B) and (D,E), revealing the outline of nuclear speckles in the presence (red outlines) and absence (white outlines) of DRB. The nuclear boundary is outlined in yellow. (I) Quantification of the projected areas in (C,F) reveal an approximate 2-fold increase in speckles size (*n* = 25 speckles, 4 cells), when transcription is inhibited by DRB.

Next, we compared the mobility of spliceosomal components in the presence and in the absence of splicing activity. HeLa cells were treated with 5,6-dichlorobenzimidazole riboside (DRB), a drug that inhibits elongation, causing premature transcriptional termination [29]. DRB is a nucleoside analog that inhibits the protein kinases that phosphorylate the C-terminal domain (CTD) of the largest subunit of RNA polymerase II in vitro [30] and in vivo [31]. Time-lapse analysis of cells expressing GFP-U2AF^65^ revealed a rapid redistribution of molecules induced by DRB (Figure 2A and 2B, and see Video S1). In less than 10 min after addition of the drug to the medium, the GFP-U2AF^65^ fluorescence decreased in the nucleoplasm and accumulated in bigger and rounder nuclear speckles. The fluorescence intensities in the nuclear speckles and in the nucleoplasm were measured, and the corresponding ratio was calculated over time (Figure 2G). Whereas in untreated cells, the ratio was 1.27 ± 0.07 (*n* = 23 speckles, 5 cells), after DRB treatment the ratio increased to 1.42 ± 0.08 (*n* = 25 speckles, 4 cells). In parallel, the average area fold increase of each nuclear speckle was measured to be 2.05 ± 0.81 (*n* = 25 speckles, 4 cells; Figure 2I). This effect was completely reverted after removal of the drug (Figure 2D, 2E, and 2H, and see Video S2). Despite a decrease in the relative fluorescence intensity, splicing proteins were still significantly detected in the nucleoplasm of DRB-treated cells, indicating that spliceosomal components can localize to this compartment even when the bulk synthesis of mRNAs is inhibited. Our quantitative estimates further suggest that a larger pool of splicing proteins localizes to nuclear speckles in DRB-treated cells, consistent with the view that spliceosomal components are targeted to the speckles when not actively engaged in splicing. As more splicing proteins localize to nuclear speckles, the projected area of these structures become approximately 2-fold larger, whereas the relative concentration of the molecules within the compartment increases only by a factor of 1.12. Thus, the density of binding sites for splicing proteins at the nuclear speckles increases only marginally in response to DRB treatment.

**Figure 3:**
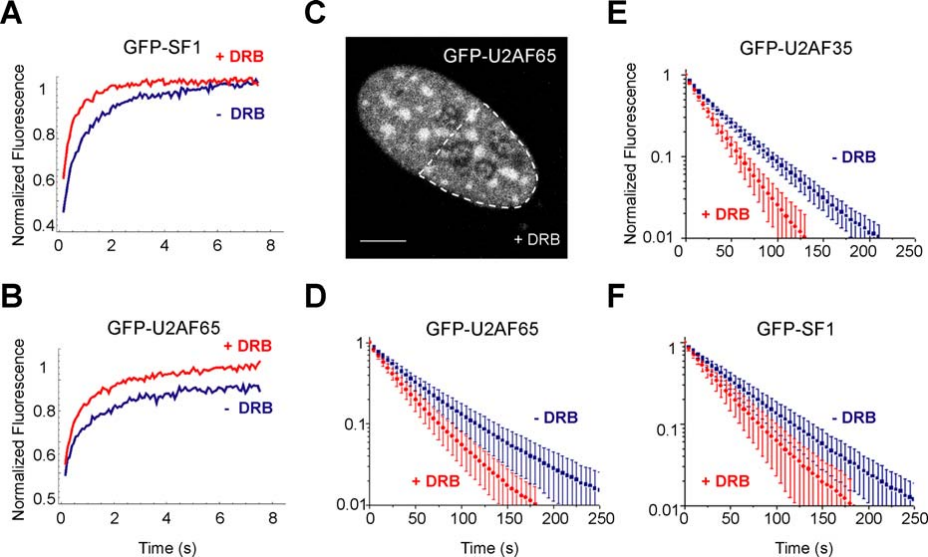
Splicing Proteins Are More Mobile in Cells Treated with DRB FRAP (A,B) and FLIP (D–F) experiments were performed on cells mock-treated (−DRB) or treated (+DRB) with DRB for 30 min. The GFP-tagged splicing proteins are indicated on each graph. For FRAP experiments, the bleached region was localized on a nuclear speckle. For FLIP experiments, the bleach region corresponded to half of the cell nucleus (dashed outline in (C), and the fluorescence decay was analyzed over a nuclear speckle. Each recovery or decay curve corresponds to a pool of three independent experiments, with ten different cells analyzed per experiment. Error bars represent standard deviations. The differences observed in the FLIP curves between DRB treated and untreated cells are statistically significant (*p* < 0.0001 for GFP-U2AF^65^ and GFP-U2AF^35^, and *p* < 0.005 for SF1).

What controls the trafficking of spliceosomal components in the nucleus is controversial. One possibility is that splicing proteins are constantly diffusing in and out of each subnuclear compartment; alternatively (or additionally), splicing proteins may receive signals to enter or leave a particular compartment. To start addressing this issue, we compared the kinetics of splicing proteins in the nucleus of HeLa cells that were either mock-treated or treated with DRB for 30 min. The results of FRAP experiments showed that none of the proteins tested was significantly immobilized in response to the drug; on the contrary, the recovery rate tended to be faster in DRB-treated cells (Figure 3A and 3B). It is noteworthy that the apparent immobile fraction of GFP-U2AF^65^ is no longer detected after DRB treatment (Figure 3B), suggesting that the transient immobilization of this splicing protein in the speckles requires ongoing transcription.

The observed FRAP results imply that, irrespective of the drug treatment, unbleached splicing proteins are constantly moving into the bleached speckles, replacing bleached molecules that moved out in the meantime. A more direct demonstration that splicing proteins continue to move out of the nuclear speckles in the absence of newly synthesized pre-mRNA was obtained by fluorescence loss in photobleaching (FLIP). A high-intensity laser was used to irreversibly destroy the GFP fluorescence in an area that corresponded to half of the cell nucleus (Figure 3C). The same area was repeatedly bleached while the loss of fluorescence in a non-bleached speckle was monitored. Experiments performed on HeLa cells treated with DRB and expressing GFP-U2AF^65^, GFP-U2AF^35^, and GFP-SF1 yielded a faster fluorescence loss in the speckles when compared to untreated cells, and quantification of fluorescence intensities in nuclear speckles over time consistently revealed significantly faster kinetics (Figure 3D–3F). We therefore conclude that shuttling of spliceosomal components between the nuclear speckles and the nucleoplasm is independent from ongoing pre-mRNA synthesis. Furthermore, the faster fluorescence recovery and decay detected by FRAP and FLIP experiments in cells treated with DRB suggests that splicing factors move more freely throughout the nucleus in the absence of transcription and splicing. Possibly, this is because the proteins spend less time bound to spliceosomes.

### A Dominant-Negative Variant of Snurportin1 Blocks Splicing and Redistributes Spliceosome Components

**Figure 4:**
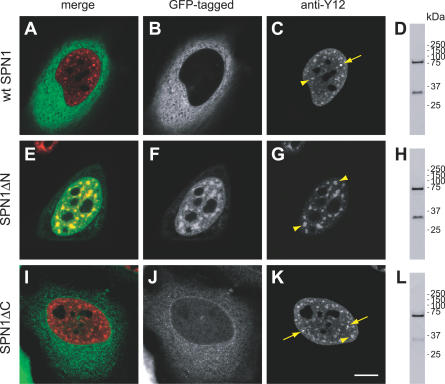
A Deletion Variant of Snurportin 1 Affects the Subnuclear Distribution of snRNPs HeLa cells were transfected with either wt snurportin1 (wt SPN1) (A–D) or the deletion variants SPN1ΔN (E–H), and SPN1ΔC (I–L) fused to GFP. The cells were labeled with mAb Y12 directed against Sm proteins. (A,E,I) Depict the superimposition of red (Y12 antibody staining) and green (GFP-tagged protein) images. (B,C,F,G,J,K) Depict the same cell imaged for GFP-tagged protein and Y12 staining, as indicated; arrows point to CBs and arrowheads to nuclear speckles. Bar indicates 10 μm. (D,H,L) Show the western blot analysis of each fusion protein. Molecular weight markers (kDa) are indicated on the left.

**Figure 5:**
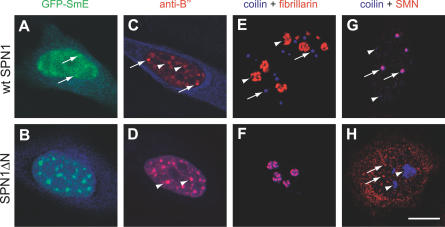
Expression of SPN1ΔN Induces Disassembly of Cajal Bodies and Enlargement of Speckles HeLa cells were transfected with either wt snurportin1 (wt SPN1) (A,C,E,G) or the deletion variant SPN1ΔN (B,D,F,H). Cells were imaged for green, red, and blue fluorescence. (A) Cell cotransfected with GFP-SmE (green) and CFP-wt SPN1 (blue). Arrows point to CBs. (B) Cell cotransfected with GFP-SmE (green) and CFP-SPN1ΔN (blue). Superimposition of green and blue shows a perfect colocalization of SmE and SPN1ΔN in round and enlarged speckles. (C) Cell transfected with CFP-wtSPN1 (blue) and immunolabeled with the antibody 4G3 directed against the U2 snRNP B′′ protein (red). Arrows point to CBs and arrowheads to nuclear speckles. (D) Cell transfected with CFP-SPN1ΔN (blue) and immunolabeled with the antibody 4G3 (red). Superimposition of red and blue images shows colocalization of snRNPs and SPN1ΔN in enlarged speckles (arrowheads). (E) Cell transfected with GFP-wtSPN1, double-labeled with antibodies directed against coilin (blue) and fibrillarin (red); arrows point to CBs and arrowheads to nucleoli. (F) Cell transfected with GFP-SPN1ΔN, double-labeled with antibodies directed against coilin (blue) and fibrillarin (red). Note that coilin relocalized from CBs to the nucleolus. (G) Cell transfected with GFP-wtSPN1, double-labeled with antibodies directed against coilin (blue) and SMN protein (red). Although SMN is detected both in the cytoplasm and in nuclear foci, the cytoplasmic staining is not well-visualized because this image was focused on CBs (arrows), which were in a confocal plane distinct from the cytoplasm. Arrowheads indicate additional minor coilin bodies that are apparently devoid of SMN. (H) Cell transfected with GFP-SPN1ΔN, double-labeled with antibodies directed against coilin (blue) and SMN (red); arrowheads point to nucleoli that now accumulate coilin and arrows point to nuclear SMN foci. Bar indicates 10 μm.

Because drugs such as DRB can cause multiple effects on cells, we thought to use an alternative approach to inhibit splicing activity. We took advantage of snurportin1 (SPN1), a nuclear import adaptor that recognizes the 2,2,7-trimethylguanosine (m_3_G) cap of spliceosomal snRNAs [10,11,32]. SPN1 is composed of two domains, an N-terminal domain required for binding to the import receptor importin-β, and a C-terminal m_3_G-cap binding region. The importin-β binding (IBB) domain comprises amino acids 1–65, and a deletion mutant lacking these residues (SPN1ΔN) retains full m_3_G-cap binding activity, but slows down or blocks the nuclear import of snRNAs [10]. Thus, SPN1ΔN appears to compete efficiently with endogenous SPN1 for binding to the m_3_G cap of the spliceosomal snRNPs. Deletion of amino acids 1–65 further prevents binding of the export receptor CRM1 to SPN1ΔN [33]. As binding of SPN1 to either CRM1 or m_3_G cap is mutually exclusive [33], the SPN1ΔN mutant most likely binds irreversibly to spliceosomal snRNPs in a dominant-negative way. To test this possibility, we fused either GFP or the cyan fluorescent protein (CFP) in frame to the amino terminus of wild-type (wt) SPN1 and SPN1ΔN. Although SPN1 is constantly shuttling between the nucleus and the cytoplasm, at steady state, the protein was predominantly detected in the cytoplasm (Figure 4A and 4B). In contrast, SPN1ΔN appeared concentrated in the nucleus (Figure 4E and 4F). A deletion mutant of snurportin1 lacking the residues 286–360 (SPN1ΔC) showed the same distribution as the wt protein (Figure 4I and 4J), arguing that the changes observed for SPN1ΔN distribution were not a consequence of the smaller size of this protein when compared to wt SPN1. Double-labeling experiments further revealed that in cells expressing wt SPN1, the Sm proteins were normally detected throughout the nucleoplasm, in nuclear speckles, and in CBs (Figure 4C), whereas in cells expressing SPN1ΔN, the Sm proteins accumulated in enlarged speckles and no longer concentrated in CBs (Figure 4G). A similar result was obtained in cells expressing GFP-SmE (Figure 5A and 5B), and after immunostaining with an antibody against the U2 snRNP protein B′′ (Figure 5C and 5D). As shown in Figures 4G, 5B, and 5D, the SPN1ΔN mutant colocalized with snRNPs in the nucleoplasm and in enlarged speckles, consistent with the assumption that it binds irreversibly to the m_3_G cap of snRNAs and fails to be recycled to the cytoplasm. Failure of SPN1ΔN to shuttle to the cytoplasm was indeed confirmed by lack of fluorescence loss in the nucleus after repeated bleaching of the cytoplasm (Figure S2).

As snRNP proteins were no longer concentrated at CBs in cells that expressed the SPN1ΔN variant, immunofluorescence was performed using antibodies against coilin. In non-transfected cells or in cells expressing wt SPN1, coilin was preferentially detected as bright foci that correspond to CBs (Figure 5E, blue foci); although CBs contain fibrillarin, this protein was observed mostly enriched at the dense fibrillar component of the nucleolus (Figure 5E, red structures). Expression of SPN1ΔN caused a major relocalization of coilin, which was no longer detected as bright foci, but rather accumulated in the nucleolus in association with fibrillarin (note superimposition of blue and red staining at nucleoli in Figure 5F). Another major component of CBs in normal cells is the SMN protein. As shown in Figure 5G, double-labeling of HeLa cells expressing wt SPN1 revealed complete colocalization of SMN and coilin at CBs (indicated by arrows; note additional minor coilin bodies that are apparently devoid of SMN, arrowheads). Following expression of SPN1ΔN, SMN dissociated from coilin (Figure 5H); SMN was still detected in foci (Figure 5H, arrows) and was never observed in nucleoli.

In conclusion, the results show that SPN1ΔN induces a redistribution of spliceosomal components similar to that observed when cells are treated with transcription inhibitors. To determine whether the mutant affected transcriptional activity, cells expressing SPN1ΔN were incubated with 5-fluorouridine (FU) for 15 min. Living cells incorporate FU into nascent RNA rapidly and specifically [34]. As previously described, nascent RNA was detected as small foci throughout the nucleoplasm and as larger structures in the nucleolus. Similar results were observed in cells transfected with either wt SPN1 or SPN1ΔN (Figure S3), indicating that the mutant does not inhibit bulk transcriptional activity.

To examine the effect of SPN1ΔN expression on splicing activity, we have used a reporter minigene transiently expressed in HeLa cells. We have shown previously that transcripts from this minigene are efficiently spliced in vivo [35]. Cells were cotransfected with SPN1ΔN constructs together with the reporter plasmid, and the transcripts derived from the reporter were analyzed 24 h post-transfection by reverse transcription followed by PCR (RT-PCR). The control cells showed three bands of approximately 375, 257, and 129 bp, as previously described [35]. The 375-bp band corresponds to the unprocessed primary transcript; the 257-bp and the 129-bp bands result from the two alternatively spliced transcripts obtained. The expression of wt SPN1 or SPN1ΔC does not affect splicing of the reporter primary transcript (Figure S4, lanes 6 and 8). By contrast, in cells expressing the variant SPN1ΔN, there is a significant reduction of spliced forms relative to unprocessed primary transcript (Figure S4, lane 4). Taken together, these results indicate that the dominant-negative SPN1ΔN mutant impairs splicing without affecting transcription.

### The Mobility of Splicing Factors Is Higher in Cells That Express SPN1ΔN

**Figure 6:**
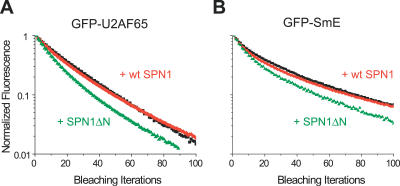
Expression of SPN1ΔN Affects the Mobility of Splicing Proteins in Living Cells FLIP experiments were performed on cells expressing GFP-U2AF^65^ (A) and GFP-SmE (B) together with the wt (+wt SPN1, red curves) or the dominant-negative mutant (+SPN1ΔN, green curves) forms of snurportin1. The fluorescence decay was analyzed over nuclear speckles. The results obtained for GFP-U2AF^65^ and GFP-SmE in cells that were not transfected with any SPN1 construct are also shown (black curves). Each decay curve corresponds to a pool of three independent experiments, with ten different cells analyzed per experiment.

Having established that expression of SPN1ΔN inhibits splicing in vivo, we next analyzed the mobility of splicing proteins in cells that express this dominant-negative peptide. HeLa cells were cotransfected with CFP fused to either wt SPN1 or SPN1ΔN and GFP-tagged splicing proteins. FLIP experiments were performed by repeatedly bleaching approximately half of the cell nucleus and monitoring the loss of fluorescence in nuclear speckles over time. The results show that the fluorescence decay curves were significantly faster (*p* < 0.0001) in cells expressing SPN1ΔN (Figure 6), similar to what was observed in cells treated with DRB (Figure 3). Taken together, the results obtained by two independent approaches show that the mobility of splicing proteins in the nucleus increases when splicing is inhibited.

### Mathematical Modeling of Splicing Factor Kinetics

Previous studies proposed that splicing proteins move within the cell nucleus by simple diffusive processes [36,37], and that transient interactions determine the steady-state subnuclear distribution of these molecules [38]. We applied a mathematical model to test whether such a combination of diffusion and binding events could explain our experimental results. In the proposed model, we assume that splicing factors constantly roam the nucleus and make transient interactions with immobile targets. In the nucleoplasm, we considered that the major binding targets are the intron-containing nascent transcripts, whereas in the speckles, the molecular nature of binding sites remains unclear.

Quantitative FRAP analysis of GFP-U2AF^65^ in HeLa cells treated with DRB revealed a diffusion rate of 1.58 μm^2^ s^−1^. Assuming that in DRB-treated cells, splicing proteins no longer assemble into spliceosomes (due to lack of newly synthesized pre-mRNA), we considered this value as representative of the effective diffusion coefficient of a splicing factor largely unaffected by binding to nascent transcripts.

Binding reactions with either nascent pre-mRNA molecules or nuclear speckles slow down the apparent diffusion rate of splicing proteins by a factor 1+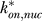 /*k_off,nuc_* [39], where 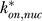 is the pseudo on-rate constant in the nucleoplasm, and *k_off,nuc_* the off-rate constant for binding sites in the nucleoplasm. The pseudo on-rate is by definition 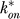 = *k_on_* S, where *k_on_* is the second-order association constant for the binding reaction and *S* is the concentration of vacant binding sites [39], which is assumed to remain constant. Because 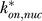 and *k_off,nuc_* cannot be directly estimated from photobleaching experiments, we empirically selected values that resulted in FRAP and FLIP simulations consistent with the experimental data. For untreated cells, we used *k_off,nuc_* = 10 s^−1^ and 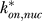 = 3.28 s^−1^. As FRAP analysis of GFP-U2AF^65^ showed a slower recovery of the fluorescence in nuclear speckles compared to the nucleoplasm (Figure 1B), the *k_off,spk_* (i.e., the off-rate constant for binding sites in the speckles) was set to 0.066 s^−1^ to achieve similar recovery rates in FRAP simulations (∼15 s to recover 90% of the initial fluorescence). The *k_on,spk_* value was then chosen to achieve a ratio between steady-state concentration of splicing proteins in the nucleoplasm and nuclear speckles similar to the experimental data (see Figure 2). As a result, the affinity (the ratio between the pseudo on- and off-rates) of splicing proteins to nuclear speckles was assigned a higher value than the affinity to nascent transcripts in the nucleoplasm (i.e., *k_off,spkn_/* 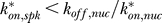 ).

To test the simulations, we further generated pure-diffusion FRAP curves, from circular bleaching regions, with different diffusion coefficients. The radial fluorescence profiles from the first post-bleach images were obtained and analyzed as previously described [40], yielding values for the effective diffusion coefficient and immobile fraction. The estimated diffusion coefficients were in good agreement with the original parameters defined for the simulations, showing that the Brownian motion algorithm was correctly implemented.

**Figure 7:**
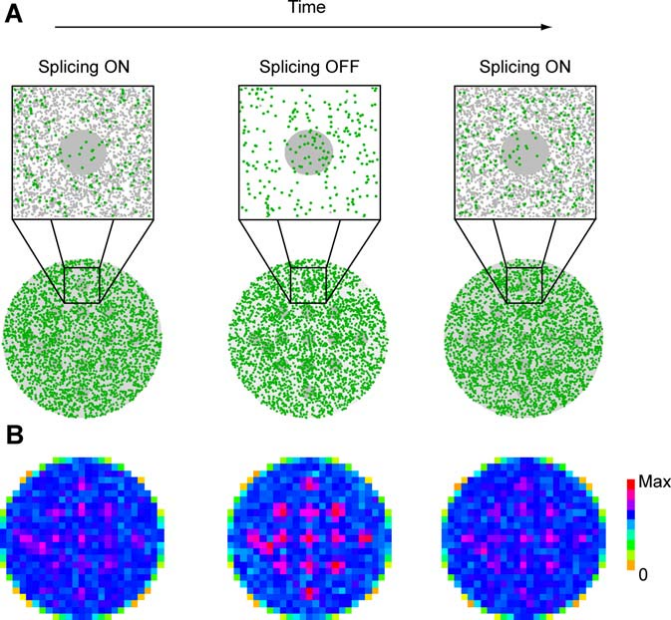
Modeling Splicing Protein Kinetics in the Cell Nucleus (A) The scheme illustrates the steady-state distribution of splicing proteins when splicing is either active (Splicing ON) or inactive (Splicing OFF). Splicing factors are represented in green and nuclear binding sites in grey. The lower row depicts positions inside the whole nucleus, and the upper row shows a zoom of the area delimited by the dashed square. The large grey circle in the upper row represents a nuclear speckle, and grey dots in the nucleoplasm represent intron-containing nascent transcripts. Note the larger number of splicing factors inside the nuclear speckle when splicing is inhibited. The parameters used for binding reactions were 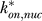 = 3.28 *s* ^−1^, *k_off,nuc_* = 10 s*^−^* ^1^, 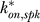 = 0.045 s^−1^, and *k_off,spk_* = 0.066 s*^−^* ^1^ (Splicing ON). To model splicing inhibition, we assumed a decrease in available nucleoplasmic binding sites; accordingly, the pseudo on-rate in the nucleoplasm was decreased by a factor of approximately 30,000, 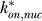 = 10^−4^ *s* ^−1^. (B) Color-code representation of the concentration of splicing factors throughout the nucleoplasm and within nuclear speckles (pink squares define dimensions and number). The simulation space corresponding to the nucleus was divided into squares (25 × 25 pixels), and the number of splicing proteins inside each square was computed (Max is the maximum value obtained in the simulations). After splicing inhibition, the number of splicing proteins at nuclear speckles is higher. Consequently, there is an increase in the local concentration (the pale pink squares become dark red) as well as in the total area occupied by highly concentrated splicing proteins (pink/red squares); this closely mimics the enlarged nuclear speckles observed in HeLa cells treated with DRB (Figure 2B and 2D) or expressing SPN1ΔN (Figure 4G).

**Figure 8:**
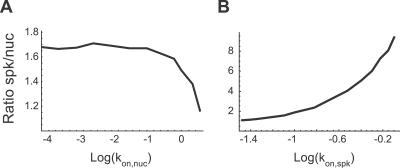
Influence of 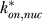
and 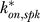
on the Relative Concentration of Splicing Proteins in Nuclear Speckles The number of splicing proteins in a speckle and in an equivalent nucleoplasmic area was counted and their ratio calculated. (A) The graph plots the ratio as a function of 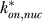 . The ratio increases with decreasing 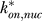 values, but stabilizes below a threshold of approximately 10^−1.5^ s^−1^. (B) The graph plots the ratio as a function of 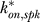 . This ratio increases unbounded with increasing 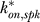 values.

A reduction of the concentration of available binding sites results in a lower probability for the binding reaction to occur. Such a reduction occurs in the nucleoplasm when splicing is inhibited. Therefore, to model splicing inhibition, we decreased 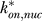 while maintaining all other parameters unaltered. This change was sufficient to cause an increase in the concentration of splicing proteins at nuclear speckles, as observed experimentally (Figure 7). The increase in concentration of splicing proteins at nuclear speckles increases with decreasing 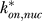 values, stabilizing below a certain value (Figure 8A). For the parameters used in the simulations, maximum concentrations were obtained with, at least, a 100-fold decrease in 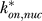 .

Theoretically, an increase in the number of splicing proteins that at steady state localize in nuclear speckles would also be expected if the affinity of these molecules to the speckles increased in response to splicing inhibition. This was in fact observed by plotting the calculated ratio between the steady-state concentration of splicing factors in the speckles and in the nucleoplasm for increasing values of 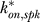 (Figure 8B).

**Figure 9:**
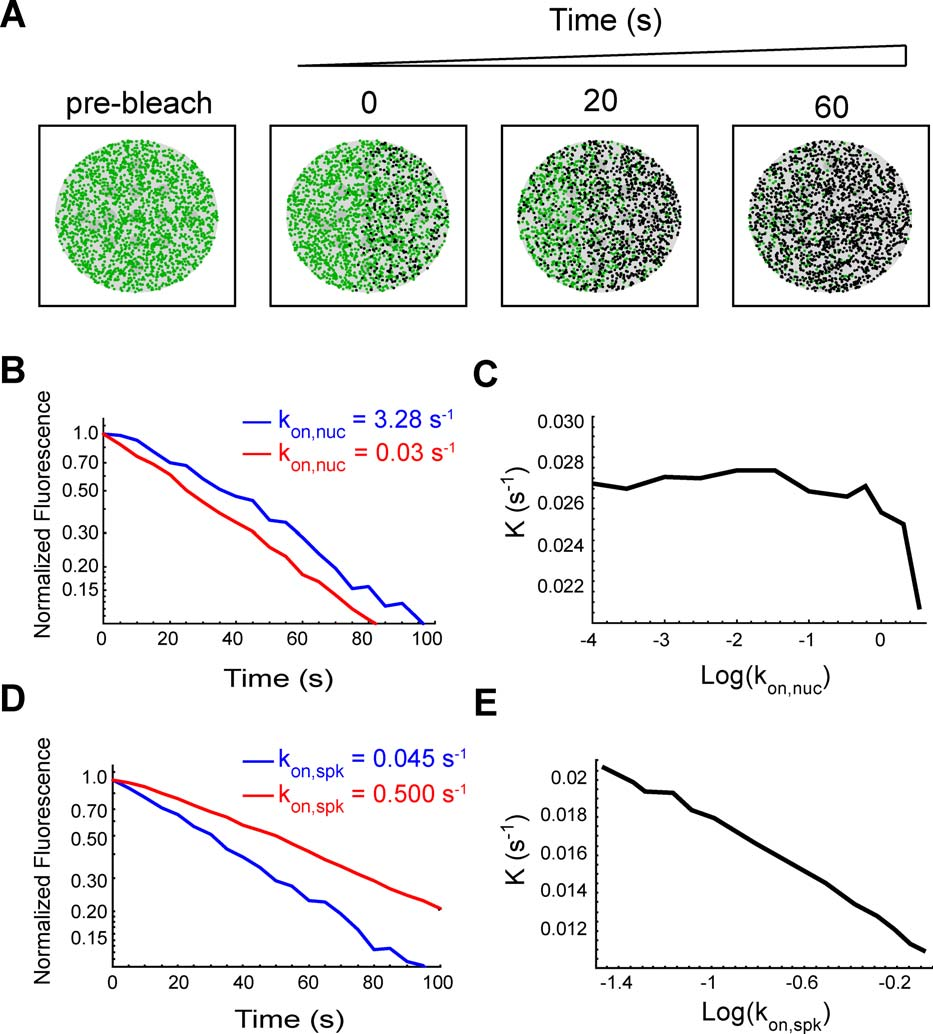
FLIP Simulations (A) Schematic illustration of a simulated FLIP sequence. The green dots represent the positions of unbleached molecules, while the black dots represent the bleached ones. The area that was repeatedly bleached corresponds to the first and the fourth quadrant of the circle that defines the nucleus. The diffusion model was run for an initial 100 s to achieve a steady state, and then a cycle of repetitive bleaching events was started. Fluorescence was monitored in the unbleached portion of the circle, both in a nuclear speckle and in a nucleoplasmic region of the same size, and at the same distance from the bleached region. (B) FLIP decay curves were generated by counting the number of fluorescent molecules inside the monitored regions at defined time intervals. For normalization, these values are divided by the number of fluorescent molecules in those regions immediately before bleaching. Normalized FLIP curves are then fitted by an exponential function of the form: *f*(*t*) = exp(−*Kt*), where *K* is the rate of fluorescence decay. Only FLIP decay curves in the nuclear speckles are depicted. The model parameters were as follows: 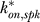 = 0.045 *s* ^−1^ , *k_off,spk_* = 0.066 *s^−^* ^1^, *k_off,nuc_* = 10 *s^−^* ^1^, and 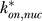 = 3.28 s^−1^ (blue line) or 0.03 s^−1^ (red line). The decay is faster for the lower 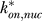 . (C) Plot of FLIP decay rates (*K*) as a function of 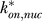 . The decay is faster for lower 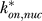 values and stabilizes below approximately 10^−1.5^ s^−1^, which implies at least a 100-fold reduction in the concentration of nucleoplasmic binding sites. (D) The effect of increasing 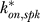 . FLIP decay curves in the speckles was obtained with the following parameters: 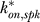 = 0.045 *s* ^−1^ (blue line) or 0.5 s^−1^ (red line), *k_off,spk_* = 0.066 s*^−^* ^1^, *k_off,nuc_* = 10 s*^−^* ^1^, and 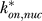 = 3.28 s^−1^. The decay is slower for the higher 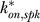 . (E) Plot of FLIP decay rates (*K*) as a function of 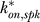 . This decay is increasingly slower for the higher values of 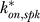 .

To further test which parameter best describes the observed kinetics after splicing inhibition (i.e., decreased 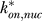 , increased 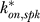 , or a combination of both), we performed FLIP simulations (Figure 9). The rate of fluorescent decay under normal conditions was very similar to the experimental data, as expected, taking into account that the simulation parameters were chosen to be consistent with the microscopic observations (Figure 3). Introducing a decrease in 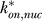 resulted in increased rates of fluorescence loss from unbleached speckles (Figure 9B), whereas increasing the 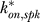 had the opposite effect (Figure 9D). Thus, decreasing the number of nucleoplasmic binding sites in the model parameters is sufficient to reproduce the faster kinetics of splicing proteins observed in cells when splicing is inhibited. Furthermore, our analysis argues against the view that splicing inhibition leads to an increased affinity of splicing proteins for the nuclear speckles, as would be expected if the proteins were kept in the speckles until a signal triggered their recruitment to nascent transcripts.

## Discussion

In this report, we show that spliceosomal snRNP and non-snRNP proteins are constantly roaming the entire nucleus, moving in and out of the speckles independently of splicing activity. Our quantitative photobleaching analysis of GFP-tagged splicing proteins in living cells, and our mathematical interpretation of the data, argue against the view that spliceosomal components are stored at nuclear speckles until a signal triggers their transit to nascent transcripts. Rather, we propose that spliceosome assembly on pre-mRNAs relies on a combination of continuous diffusion and transient interactions.

To study how spliceosomal components are recruited to newly synthesized pre-mRNAs in the nucleus, we determined the mobility kinetics of splicing proteins in the presence or absence of splicing activity. FRAP and FLIP experiments were performed using a number of GFP-tagged proteins. Previous studies confirmed that chimeras of GFP fused to ASF/SF2 [21], snRNP proteins [16,41], and SC35 [42] behave similarly to the native proteins.

A classical approach to block splicing activity in vivo consists in using drugs such as actinomycin D, α-amanitin, or DRB, which primarily inhibit transcription. Here, in addition to treating cells with DRB, we thought to inhibit splicing activity by an independent mechanism. We show that expression of a dominant-negative deletion mutant of the snRNP-specific nuclear import adaptor snurportin1 (SPN1) specifically prevents splicing of a reporter minigene. SPN1 is an adaptor protein that binds simultaneously to the m_3_G-cap structure of spliceosomal snRNAs and to the nuclear import receptor importin-β [10,11,32]. Like other adaptor proteins, SPN1 shuttles continuously between the nucleus and the cytoplasm, binding cargo (snRNPs) in the cytoplasm, releasing the cargo in the nucleus, and recycling back to the cytoplasm without cargo. At steady state, SPN1 was predominantly detected in the cytoplasm (Figure 4A and 4B), indicating that the protein exits rapidly from the nucleus. SPN1 is transported out of the nucleus by the export receptor CRM1, and binding of SPN1 to either CRM1 or m_3_G cap is mutually exclusive [33]. Thus, CRM1 only exports SPN1 molecules that have already released their snRNP cargo in the nucleus. A SPN1 deletion mutant that lacks amino acids 1–65 (SPN1ΔN) retains the capacity to bind the m_3_G cap but blocks nuclear import of snRNPs because it lacks the IBB domain required for binding to and import by importin-β [10]. Moreover, deletion of amino acids 1–65 prevents binding of CRM1 to SPN1 [33] and consequently impairs export of SPN1ΔN from the nucleus (Figure S2). Because SPN1 binds very tightly to the m_3_G cap, in the absence of the competing CRM1 interaction, snRNPs are expected to remain bound to SPN1. In good agreement with this prediction, we observe a perfect colocalization of snRNPs and SPN1ΔN (Figure 4E–4G). SPN1ΔN is a small protein (<45 kDa [10]) capable of diffusing through the nuclear pore complex [43]. In the nucleus, SPN1ΔN most probably binds tightly to the m_3_G cap of snRNPs, thereby blocking spliceosome assembly. Consistent with this view, we observe that splicing of a reporter minigene is blocked in cells that express SPN1ΔN, whereas splicing is unaffected by expression of SPN1ΔC, a deletion variant that lacks the m_3_G-cap binding domain (Figure S4). Expression of SPN1ΔN further induces an accumulation of snRNPs and non-snRNP splicing proteins in enlarged nuclear speckles, similar to what is observed following splicing inhibition by treatment with transcription inhibitors [13,19] or microinjection of oligonucleotides or antibodies targeted to disrupt splicing [22]. Potential indirect effects caused by drug treatment have been excluded by showing that α-amanitin does not alter the distribution of snRNPs in the nucleus of cells that express an α-amanitin–resistant form of RNA polymerase II [44]. Thus, it is generally accepted that the accumulation of spliceosomal components in enlarged nuclear speckles occurs as a consequence of splicing inhibition.

We also show here that expression of SPN1ΔN causes the disappearance of CBs with redistribution of coilin to the nucleolus (Figure 5). Because the SPN1ΔN deletion mutant blocks nuclear import of newly synthesized snRNPs [10], this finding strengthens the view that CBs form as a result of ongoing snRNP biogenesis in the nucleus. Indeed, several recent studies reported the disappearance of CBs upon depletion of SMN, which disrupted Sm core assembly; PHAX, which blocked specifically the nuclear export of newly synthesized U snRNAs; or hTGS1, which impaired m^7^G-cap methylation [45–47]. Additionally, CBs disassemble when splicing is inhibited by treating cells with transcription inhibitors [48]. This further suggests that CBs are transient compartments, the maintenance of which requires both ongoing biogenesis of new snRNPs and continuous recycling of pre-existing snRNPs after each round of spliceosome assembly.

Previous FRAP studies using GFP-tagged ASF/SF2 and SC35 revealed that these splicing proteins are in constant flux and move throughout the entire nucleus, regardless of their initial location [37,49]. Our results show that GFP-tagged versions of SC35, SF3a120, SF1, U2AF^65^, and U2AF^35^ have diffusion rates in the nucleoplasm ranging from 1.2 to 1.4 μm^2^/s. By contrast, GFP-SmE diffuses at significantly lower rate (0.7 μm^2^/s). All proteins recovered faster in the nucleoplasm than in the speckles, but the difference is most striking for SmE, SF3a120, and U2AF^65^, which also showed apparent immobile fractions. We further show that the mobility of SmE is significantly lower in CBs than in the nucleoplasm and similar to the mobility observed in nuclear speckles (Figure 1B and 1C). The dynamic exchange of several CB components has been previously demonstrated in both mammalian cells [14,50,51] and in *Xenopus* germinal vesicles [52]. In agreement with our results, the spliceosomal snRNP core proteins SmB and SmD1 were estimated to reside in CBs for several seconds [51]. In contrast to snRNP proteins, coilin and SMN have residence times in CBs on the order of minutes, whereas the U4/U6 snRNP assembly factor SART3 dissociates from CBs after just a few seconds [51].

We observed that all spliceosomal proteins are continuously shuttling between the nucleoplasm and nuclear speckles, or between CBs, nucleoplasm, and nuclear speckles, and the exchange process occurs on the order of seconds. According to our previous estimates, GFP (27 kDa) diffuses in cells at 33 μm^2^ s^−1^ [40], and an unbound GFP-fusion protein with approximately 60 KDa is expected to diffuse at approximately 25.3 μm^2^ s^−1^ [53]. The significantly lower diffusion rates estimated for splicing proteins (Figure 1) suggest that these molecules are slowed down by interactions with less-mobile nuclear components. Furthermore, the slower recovery observed in nuclear speckles and CBs relative to the nucleoplasm could be due to lower mobility of splicing proteins inside these compartments, transient immobilization caused by binding to fixed structures, or a combination of both. How long a splicing protein resides in a particular compartment depends on its binding affinity to interacting molecules located in that compartment, and the binding kinetics may change over time in response to specific signals. According to the latter view, the accumulation of spliceosomal components in enlarged nuclear speckles following transcription and/or splicing inhibition could result from a longer retention at the speckle compartment. However, the results of photobleaching experiments depicted in Figures 3 and 6 argue against that possibility. Our FLIP data clearly demonstrate that spliceosomal proteins are constantly diffusing away from nuclear speckles independently of whether splicing is active or inactive, and the rate of fluorescence loss from the speckles increased after splicing inhibition. In agreement with these results, it was previously reported that the nuclear mobility of GFP-ASF increased after treating the cells with transcriptional inhibitors [37].

The finding that inhibition of splicing activity leads to concentration of spliceosomal proteins in enlarged nuclear speckles and yet the proteins move away from the speckles at faster rates is counterintuitive. To address this apparent paradox, we performed mathematical simulations using a model that relies exclusively on stochastic processes. In our model, splicing proteins were considered as particles moving by Brownian diffusion. The nucleoplasm and the nuclear speckles were considered as regions that provide binding sites for splicing proteins. The on- and off-rates for the binding were considered to differ in the nucleoplasm and the nuclear speckles to be consistent with the FRAP and FLIP experimental data. Inhibition of splicing, which reduces the number of pre-mRNA binding sites available for spliceosome assembly, was modeled as a decrease in the on-rate constant in the nucleoplasm. Simulation of time-lapse microscopy and FLIP experiments before and after splicing inhibition yielded results consistent with experimental observations. Indeed, the model reproduced both the accumulation of particles in speckles and the faster mobility of particles. The model assumes that under normal conditions, a diffusing particle (representing a spliceosomal protein) can either bind to a spliceosome in the nucleoplasm or to unknown partners in the speckles. In a simulated steady-state situation, this results in 11% of the particles being bound to the speckles while 22% are bound to spliceosomes in the nucleoplasm. The remaining 67% are freely diffusing in the nucleus. By decreasing the number of binding sites in the nucleoplasm, splicing inhibition reduces the binding competition between speckles and nucleoplasm. Consequently, a particle has higher chances of binding to a speckle, becoming temporarily part of it. As more particles bind simultaneously to nuclear speckles, their concentration increases and the compartment enlarges. In parallel, there are more particles diffusing because they are no longer retained by spliceosomes in the nucleoplasm. In the simulations, the percentage of diffusing particles increases to 86% after the number of binding sites in the nucleoplasm is reduced. Because there are more particles diffusing, there is a higher chance that they will reach the bleach region, resulting in faster loss of fluorescence.

In conclusion, we show that the changes in kinetic behavior observed for spliceosomal proteins following inhibition of splicing can be reproduced in a stochastic model by simply reducing the on-rate for binding to spliceosomes in the nucleoplasm. Our simulations further demonstrate that increasing the on-rate for binding to the speckles would decrease the rate of fluorescence loss from that compartment, as opposed to the FLIP results obtained experimentally. Thus, it is unlikely that spliceosomal components require a splicing-dependent signal in order to leave the nuclear speckles. Rather, we favor the view that splicing proteins are constantly diffusing throughout the entire nucleus and collide randomly and transiently with either pre-mRNAs or nuclear speckle components.

## Materials and Methods

### Cell culture, transfection, and drug treatment.

Human HeLa cells (ECACC 93021013) were grown as monolayers in minimum essential medium with Earle's salts (DMEM) supplemented with 10% (v/v) fetal calf serum, 1% (v/v) nonessential amino acids (Gibco, Invitrogen), and 2 mM L-glutamine (Gibco, Invitrogen). For live imaging, the cells were plated in glass-bottom chambers (MatTek), and the medium was changed to D-MEM/F-12 without phenol red, supplemented with 15 mM HEPES buffer (Invitrogen). Subconfluent cells were transfected with FuGENE6 reagent (Roche Biochemicals) and analyzed at 16–24 h after transfection. The transcription inhibitor DRB (Sigma) was used at 75 μM from a stock solution of 11 mM in ethanol.

### In vivo transcription and splicing assays.

For in vivo analysis of transcriptional activity, cells were incubated for 15 min in cell culture medium supplemented with 2 mM 5′-fluoruridine (5'-FU; Sigma-Aldrich). The incorporated 5'-FU residues were detected with a mouse monoclonal antibody anti-BrdU (clone BU-33; Sigma). For splicing analysis, we used the reporter plasmid IgM-Minx, which is a chimera of IgM and AdML splicing substrates, as previously described [35]. HeLa cells growing on 35-mm Petri dishes were cotransfected with 1 μg of GFP-SPN1 constructs plus 200 ng of the reporter plasmid.

### Immunofluorescence and western blotting.

For indirect immunofluorescence, cells were washed twice in PBS, fixed with 3.7% formaldehyde in PBS for 10 min at room temperature, and subsequently permeabilized with 0.5% Triton X-100 in PBS for 15 min at room temperature. The cells were then rinsed in PBS containing 0.05% Tween-20 (PBS-Tw), incubated for 60 min with primary antibodies diluted in PBS, washed in PBS-Tw, and incubated for 30 min with the appropriate secondary antibodies conjugated to fluorescein (FITC), indocarbocyanine (Cy3), or indodicarbocyanine (Cy5) (Jackson ImmunoResearch Laboratories). Finally, the coverslips were mounted in VectaShield (Vector Laboratories) and sealed with nail polish.

We used antibodies directed against the following proteins: Sm (mAb Y12; [54]), U2 snRNP protein B′′ (mAb 4G3; [55]), coilin (rabbit serum 204.3, kindly provided by Professor A. Lamond, University of Dundee, United Kingdom), and fibrillarin (mAb 72B9; [56]) and SMN (mAb 2B1, a gift from Professor G. Dreyfuss, University of Pennsylvania, Philadelphia, Pennsylvania, United States).

For western blotting analysis, protein extracts were prepared by scraping the cells into SDS-PAGE buffer (40 mM Tris-HCl [pH 6.8], 8% glycerol, 2.4% SDS, 75 mM DTT, 0.01% bromophenol blue) with 200 U/ml benzonzse (Sigma-Aldrich), incubating for 10 min at room temperature and then boiling for 5 min. Volumes of total protein extract equivalent to 5 × 10^5^ cells were separated on 10% SDS-polyacrylamide gels and transferred to nitrocellulose membranes. Western blotting was carried out using a semidry electrophoretic transfer cell. Blots were probed with anti-GFP monoclonal antibody (Roche Applied Sciences) in 2.5% milk-PBS and developed using peroxidase-conjugated goat anti-mouse IgG (BioRad Laboratories). Bands were visualized using ECL (Amersham Biosciences).

### Plasmids and constructs.

The following GFP-tagged proteins were used: GFP-SmE (kindly provided by Professor A. Lamond), GFP-SC35 (a gift from Dr. Jan-Peter Kreivi, Uppsala University, Sweden), GFP-U2AF^65^ and GFP-U2AF^35^ [57], GFP-SF3a120 (a gift from Professor Angela Krämer, University of Geneva, Switzerland), and GFP-SF1. The cDNA of SF1 (Y08766) was obtained from pGEM/SF1 (a gift from Professor Angela Krämer) and cloned in the Bam HI site of pEGFP-C1 (Clontech). The pET28 snurportin1 constructs [10] were digested with Nco I, followed by a fill-in reaction, digestion with Bam HI, and cloning into the Sma I and Bam HI sites of pECFP-C1, pEGFP-C1, and pEGFP-N3 vectors. All constructs were purified using plasmid DNA midi-prep kit (QIAGEN) and sequenced.

### Live cell microscopy.

Live cells were imaged at 37 °C maintained by a heating/cooling frame (LaCon,) in conjunction with an objective heater (PeCon). Images were acquired on a Zeiss LSM 510 confocal microscope (Carl Zeiss) using a PlanApochromat 63×/1.4 objective. GFP fluorescence was detected using the 488-nm laser line of an Ar laser (25 mW nominal output) and a LP 505 filter. Time-lapse 3D imaging of selected cells was performed on the confocal microscope immediately after DRB treatment and/or DRB removal. For this, a total of up to 200 *z*-stack series were acquired over time for each cell, each *z*-stack having between 15 and 20 images and with 0.60 μm of distance between each image in the stack. Image size was 512 × 512 pixels, and the pixel width was 72 nm. The time between each *z*-stack acquisition depended on its number of images, and varied between 20 s and 60 s. Maximum projection images were generated from each *z*-stack and processed with ImageJ (http://rsb.info.nih.gov/ij/) using a rigid body registration algorithm to correct for cell displacement during image acquisition. Movies of cells after treatment or removal of DRB were then generated and time-annotated. Fluorescence intensity values in nuclear speckles and nucleoplasmic regions were measured over time in registered projection images also using ImageJ.

### FLIP analysis.

In each FLIP experiment, cells were repeatedly bleached in a region of interest (ROI) that corresponded to half of the total nuclear area, and imaged between bleach pulses. Bleaching was performed by scanning the defined ROI with three iterations of the 488-nm laser line, at maximum intensity. Bleach pulse duration ranged from 2.2 to 3.1 s, depending on the size of the bleached region. Repetitive bleach pulses were achieved using the FLIP Macro for LSM software release 2.8, developed by Gwénaël Rabut, at the EMBL (http://www.embl-heidelberg.de/ExternalInfo/ellenberg/homepage/macros.html). Image size was 512 × 512 pixels, and the pixel width was 48 nm. For imaging, the laser power was attenuated to 0.1%–0.2% of the bleach intensity. Images were background subtracted and registered to correct for cell displacement during image acquisition using ImageJ. Fluorescence intensity values in nuclear speckles and nucleoplasmic regions were measured over time in registered projection images using ImageJ. The data were then normalized to correct for loss of fluorescence due to image acquisition, using non-bleached cells to estimate imaging bleach kinetics. Loss of fluorescence due to imaging could reach 10%–20% over the time course of the experiment.

### Quantitative FRAP analysis.

FRAP experiments were performed essentially as described [40]. Each FRAP experiment started with three image scans followed by a bleach pulse of 110 ms on a spot with a diameter of 25 pixels (0.59-μm radius). A series of 97 single-section images (of size 512 × 50 and pixel width 48 nm) was then collected at intervals of 78.40 ms, again with the first image acquired 2 ms after the end of bleaching. For FRAP performed during a longer time, all parameters were kept the same, except the number of images, which was increased to 997 (total duration of acquisition thus increased to ∼80 s). For imaging, the laser power was attenuated to 1% of the bleach intensity. FRAP time series were analyzed as described [40]. All fitting procedures were performed using the NonLinearRegress function of Mathematica 5.0 (Wolfram Research).

### Mathematical modeling.

In our model, the nucleoplasm and nuclear speckles were defined as circular regions with radii 8 μm and 0.7 μm, respectively, and the number of speckles was *n*_s_ = 14. The Brownian motion algorithm was based on a modification of the Box-Müller algorithm [58] to generate random deviates with Gaussian distribution and standard deviation equal to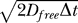 in each spatial direction. Typically Δ*t* (the time between each simulation step) was set to 0.01 s, which yields a standard deviation of approximately 0.17 μm, less than the size of a nuclear speckle. The effective diffusion coefficient was set to *D_free_* = 1.58 μm^2^ s^−1^ based on the value estimated by FRAP experiments for GFP-U2AF65 in DRB-treated cells. Binding reactions slow the apparent diffusion rate of molecules by a factor 1+ 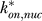 /*k_off,nuc_* [39]. The probability for the binding reaction to occur is *p_bind_* = 1 − exp 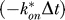 , whereas the probability for a bound molecule to break its interaction is *p*_unbind_ = 1 − exp(−*k_off_*Δ*t*). These probabilities are position-dependent, being different for the nucleoplasm and the speckles. Within the nucleoplasm and the nuclear speckles, we assumed that binding sites were distributed homogeneously. The values for *k_on_* and *k_off_* were empirically selected from FRAP and FLIP simulations.

FRAP and FLIP simulations were generated using a Monte Carlo approach. At least 10^5^ molecules were simulated. The probability that a fluorescent molecule is bleached is *p*_bleach_ = 1 − exp(−*K_B_*Δ*t*) inside the bleaching region. For FRAP simulations, the bleach region consisted of a circular area (0.7-μm radius) positioned either in the nucleoplasm or in a nuclear speckle, and for FLIP simulations, half of the circle representing the nucleus was bleached. In a simulation step, for each molecule, a random number α with uniform probability density was generated in the interval [0,1]. The random number generator used is based on an implementation (http://fmg-www.cs.ucla.edu/fmg-members/geoff/mtwist.html) of the Mersenne-Twister algorithm [59], which generates deviates with uniform distribution. For a non-bound molecule, the binding reaction occurs if α < *p_Bind_*. If α > *p_Bind_*, then the molecule moves to another random location according to the Brownian motion algorithm. Molecules are constrained to move inside the nucleus. Bound molecules maintain their current coordinates and are freed from their sites only if α < *p_unbind_*. Fluorescent molecules are bleached if the particle is located inside the bleach region and if a random number β in the interval [0,1] is smaller than *p_bleach_*. FRAP and FLIP curves were generated by counting at defined time intervals the number of fluorescent molecules inside either the bleached or unbleached regions, respectively.

## Supporting Information

Figure S1 GFP-U2AF^65^ Is Transiently Immobilized in Nuclear Speckles FRAP experiments were performed on HeLa cells expressing GFP-tagged U2AF^65^. The panel shows the FRAP recovery curve in nuclear speckles. This curve corresponds to a pool of three independent experiments, with ten different cells analyzed per experiment.
(1.2 MB TIF)

Figure S2 The Mutant SPN1ΔN Fails To Be Exported from the Nucleus FLIP experiments were performed on HeLa cells expressing GFP-wt SPN1 or GFP-SPN1ΔN. The wild-type protein is predominantly detected in the cytoplasm. However, the fluorescence in the cytoplasm decays following repeated bleaching of the nucleus, indicating that the protein shuttles between the two compartments. In contrast, the intensity of GFP-SPN1ΔN fluorescence in the nucleus remains constant after repeated bleaching of the cytoplasm, indicating that this mutant protein is not shuttling. Each decay curve corresponds to six different cells analyzed. Fluorescence values were corrected for bleaching due to imaging, using ten different unbleached cells imaged in the same conditions. Error bars represent standard deviations.
(2.4 MB TIF)

Figure S3 Expression of SPN1ΔN Does Not Affect Transcriptional Activity HeLa cells were transfected with either wtSPN1 (A–C) or the deletion mutant SPN1ΔN (D–F). Cells were incubated with 5′-fluoruridine (5'-FU) for 15 min. Living cells incorporate 5′-FU into nascent RNA, which is visualized with antibodies against halogenated nucleotides (red staining in (B,C,E,F)), showing that global transcription is active in both transfected cells. Bar indicates 10 μm.
(3.2 MB TIF)

Figure S4 Expression of SPN1ΔN Impairs Splicing RT-PCR analysis of HeLa cells that were either transfected with a splicing reporter minigene (lanes 2 and 3) or cotransfected with the reporter minigene together with GFP-SPN1ΔN (lanes 4 and 5), GFP-SPN1ΔC (lanes 6 and 7), or GFP-wtSPN1 (lanes 8 and 9). Controls lacking reverse transcriptase indicate no contamination with plasmid DNA (lanes 3, 5, 7, and 9). The structure of each transcript is illustrated on the right. Molecular weight markers (kDa) are indicated on the left.
(3.2 MB TIF)

Video S1 Live-Cell Movie Played at 750 Times Its Real Speed Showing Redistribution of GFP-Tagged U2AF^65^ after Addition of DRB to the Cell Medium In less than 10 min after addition of the drug, GFP-U2AF^65^ fluorescence decreased in the nucleoplasm and accumulated in bigger, rounder speckles.
(6.2 MB AVI)

Video S2 Live-Cell Movie Showing Redistribution of GFP-Tagged U2AF^65^ after Removal of DRB to the Cell Medium The effect of the drug as shown in Video S1 is completely reversible.
(10.3 MB AVI)

## Abbreviations

CB: Cajal body
CFP: cyan fluorescent protein
FLIP: fluorescence loss in photobleaching
FRAP: fluorescence recovery after photobleaching
GFP: green fluorescent protein
IGC: interchromatin granule cluster
SMN: survival of motor neurons
snRNA: small nuclear RNA
snRNP: small nuclear ribonucleoprotein particle
wt: wild type

## References

[pcbi-0030201-b001] Nilsen TW (2003). The spliceosome: the most complex macromolecular machine in the cell?. Bioessays.

[pcbi-0030201-b002] Jurica MS, Moore MJ (2003). Pre-mRNA splicing: awash in a sea of proteins. Mol Cell.

[pcbi-0030201-b003] Will CL, Luhrmann R (2001). Spliceosomal UsnRNP biogenesis, structure and function. Curr Opin Cell Biol.

[pcbi-0030201-b004] Achsel T, Stark H, Luhrmann R (2001). The Sm domain is an ancient RNA-binding motif with oligo(U) specificity. Proc Natl Acad Sci U S A.

[pcbi-0030201-b005] Kambach C, Walke S, Young R, Avis JM, de la Fortelle E (1999). Crystal structures of two Sm protein complexes and their implications for the assembly of the spliceosomal snRNPs. Cell.

[pcbi-0030201-b006] Stark H, Dube P, Luhrmann R, Kastner B (2001). Arrangement of RNA and proteins in the spliceosomal U1 small nuclear ribonucleoprotein particle. Nature.

[pcbi-0030201-b007] Bertrand E, Bordonne R (2004). Assembly and traffic of small nuclear RNPs. Prog Mol Subcell Biol.

[pcbi-0030201-b008] Yong J, Wan L, Dreyfuss G (2004). Why do cells need an assembly machine for RNA-protein complexes?. Trends Cell Biol.

[pcbi-0030201-b009] Filipowicz W, Pogacic V (2002). Biogenesis of small nucleolar ribonucleoproteins. Curr Opin Cell Biol.

[pcbi-0030201-b010] Huber J, Cronshagen U, Kadokura M, Marshallsay C, Wada T (1998). Snurportin1, an m3G-cap-specific nuclear import receptor with a novel domain structure. EMBO J.

[pcbi-0030201-b011] Strasser A, Dickmanns A, Luhrmann R, Ficner R (2005). Structural basis for m3G-cap-mediated nuclear import of spliceosomal UsnRNPs by snurportin1. EMBO J.

[pcbi-0030201-b012] Narayanan U, Achsel T, Luhrmann R, Matera AG (2004). Coupled in vitro import of U snRNPs and SMN, the spinal muscular atrophy protein. Mol Cell.

[pcbi-0030201-b013] Spector DL (1993). Macromolecular domains within the cell nucleus. Annu Rev Cell Biol.

[pcbi-0030201-b014] Sleeman JE, Trinkle-Mulcahy L, Prescott AR, Ogg SC, Lamond AI (2003). Cajal body proteins SMN and Coilin show differential dynamic behaviour in vivo. J Cell Sci.

[pcbi-0030201-b015] Cioce M, Lamond AI (2005). Cajal bodies: a long history of discovery. Annu Rev Cell Dev Biol.

[pcbi-0030201-b016] Stanek D, Rader SD, Klingauf M, Neugebauer KM (2003). Targeting of U4/U6 small nuclear RNP assembly factor SART3/p110 to Cajal bodies. J Cell Biol.

[pcbi-0030201-b017] Schaffert N, Hossbach M, Heintzmann R, Achsel T, Luhrmann R (2004). RNAi knockdown of hPrp31 leads to an accumulation of U4/U6 di-snRNPs in Cajal bodies. EMBO J.

[pcbi-0030201-b018] Fakan S (1994). Perichromatin fibrils are in situ forms of nascent transcripts. Trends Cell Biol.

[pcbi-0030201-b019] Lamond AI, Spector DL (2003). Nuclear speckles: a model for nuclear organelles. Nat Rev Mol Cell Biol.

[pcbi-0030201-b020] Janicki SM, Tsukamoto T, Salghetti SE, Tansey WP, Sachidanandam R (2004). From silencing to gene expression: real-time analysis in single cells. Cell.

[pcbi-0030201-b021] Misteli T, Caceres JF, Spector DL (1997). The dynamics of a pre-mRNA splicing factor in living cells. Nature.

[pcbi-0030201-b022] O'Keefe RT, Mayeda A, Sadowski CL, Krainer AR, Spector DL (1994). Disruption of pre-mRNA splicing in vivo results in reorganization of splicing factors. J Cell Biol.

[pcbi-0030201-b023] Misteli T (2007). Beyond the sequence: cellular organization of genome function. Cell.

[pcbi-0030201-b024] Carrero G, Hendzel MJ, de Vries G (2006). Modelling the compartmentalization of splicing factors. J Theor Biol.

[pcbi-0030201-b025] Soula H, Robardet C, Perrin F, Gripon S, Beslon G (2005). Modeling the emergence of multi-protein dynamic structures by principles of self-organization through the use of 3DSpi, a multi-agent-based software. BMC Bioinformatics.

[pcbi-0030201-b026] Misteli T (2001). The concept of self-organization in cellular architecture. J Cell Biol.

[pcbi-0030201-b027] Matera AG, Shpargel KB (2006). Pumping RNA: nuclear bodybuilding along the RNP pipeline. Curr Opin Cell Biol.

[pcbi-0030201-b028] Platani M, Goldberg I, Swedlow JR, Lamond AI (2000). In vivo analysis of Cajal body movement, separation, and joining in live human cells. J Cell Biol.

[pcbi-0030201-b029] Chodosh LA, Fire A, Samuels M, Sharp PA (1989). 5,6-Dichloro-1-beta-D-ribofuranosylbenzimidazole inhibits transcription elongation by RNA polymerase II in vitro. J Biol Chem.

[pcbi-0030201-b030] Serizawa H, Conaway JW, Conaway RC (1993). Phosphorylation of C-terminal domain of RNA polymerase II is not required in basal transcription. Nature.

[pcbi-0030201-b031] Dubois MF, Nguyen VT, Bellier S, Bensaude O (1994). Inhibitors of transcription such as 5,6-dichloro-1-beta-D-ribofuranosylbenzimidazole and isoquinoline sulfonamide derivatives (H-8 and H-7) promote dephosphorylation of the carboxyl-terminal domain of RNA polymerase II largest subunit. J Biol Chem.

[pcbi-0030201-b032] Huber J, Dickmanns A, Luhrmann R (2002). The importin-beta binding domain of snurportin1 is responsible for the Ran- and energy-independent nuclear import of spliceosomal U snRNPs in vitro. J Cell Biol.

[pcbi-0030201-b033] Paraskeva E, Izaurralde E, Bischoff FR, Huber J, Kutay U (1999). CRM1-mediated recycling of snurportin 1 to the cytoplasm. J Cell Biol.

[pcbi-0030201-b034] Boisvert FM, Hendzel MJ, Bazett-Jones DP (2000). Promyelocytic leukemia (PML) nuclear bodies are protein structures that do not accumulate RNA. J Cell Biol.

[pcbi-0030201-b035] Pacheco TR, Coelho MB, Desterro JM, Mollet I, Carmo-Fonseca M (2006). In vivo requirement of the small subunit of U2AF for recognition of a weak 3′ splice site. Mol Cell Biol.

[pcbi-0030201-b036] Pederson T (2000). Diffusional protein transport within the nucleus: a message in the medium. Nat Cell Biol.

[pcbi-0030201-b037] Phair RD, Misteli T (2000). High mobility of proteins in the mammalian cell nucleus. Nature.

[pcbi-0030201-b038] Misteli T (2001). Protein dynamics: implications for nuclear architecture and gene expression. Science.

[pcbi-0030201-b039] Sprague BL, Pego RL, Stavreva DA, McNally JG (2004). Analysis of binding reactions by fluorescence recovery after photobleaching. Biophys J.

[pcbi-0030201-b040] Braga J, Desterro JM, Carmo-Fonseca M (2004). Intracellular macromolecular mobility measured by fluorescence recovery after photobleaching with confocal laser scanning microscopes. Mol Biol Cell.

[pcbi-0030201-b041] Sleeman JE, Lamond AI (1999). Nuclear organization of pre-mRNA splicing factors. Curr Opin Cell Biol.

[pcbi-0030201-b042] Politz JC, Tuft RA, Pederson T (2003). Diffusion-based transport of nascent ribosomes in the nucleus. Mol Biol Cell.

[pcbi-0030201-b043] Bonner WM, Bush H (1978). Protein migration and accumulation in the nuclei. The cell nucleus.

[pcbi-0030201-b044] Custodio N, Antoniou M, Carmo-Fonseca M (2006). Abundance of the largest subunit of RNA polymerase II in the nucleus is regulated by nucleo-cytoplasmic shuttling. Exp Cell Res.

[pcbi-0030201-b045] Shpargel KB, Matera AG (2005). Gemin proteins are required for efficient assembly of Sm-class ribonucleoproteins. Proc Natl Acad Sci U S A.

[pcbi-0030201-b046] Girard C, Neel H, Bertrand E, Bordonne R (2006). Depletion of SMN by RNA interference in HeLa cells induces defects in Cajal body formation. Nucleic Acids Res.

[pcbi-0030201-b047] Lemm I, Girard C, Kuhn AN, Watkins NJ, Schneider M (2006). Ongoing U snRNP biogenesis is required for the integrity of Cajal bodies. Mol Biol Cell.

[pcbi-0030201-b048] Carmo-Fonseca M, Pepperkok R, Carvalho MT, Lamond AI (1992). Transcription-dependent colocalization of the U1, U2, U4/U6, and U5 snRNPs in coiled bodies. J Cell Biol.

[pcbi-0030201-b049] Kruhlak MJ, Lever MA, Fischle W, Verdin E, Bazett-Jones DP (2000). Reduced mobility of the alternate splicing factor (ASF) through the nucleoplasm and steady state speckle compartments. J Cell Biol.

[pcbi-0030201-b050] Snaar S, Wiesmeijer K, Jochemsen AG, Tanke HJ, Dirks RW (2000). Mutational analysis of fibrillarin and its mobility in living human cells. J Cell Biol.

[pcbi-0030201-b051] Dundr M, Hebert MD, Karpova TS, Stanek D, Xu H (2004). In vivo kinetics of Cajal body components. J Cell Biol.

[pcbi-0030201-b052] Handwerger KE, Murphy C, Gall JG (2003). Steady-state dynamics of Cajal body components in the Xenopus germinal vesicle. J Cell Biol.

[pcbi-0030201-b053] Braga J, McNally JG, Carmo-Fonseca M (2007). A reaction-diffusion model to study RNA motion by quantitative fluorescence recovery after photobleaching. Biophys J.

[pcbi-0030201-b054] Lerner EA, Lerner MR, Janeway CA, Steitz JA (1981). Monoclonal antibodies to nucleic acid-containing cellular constituents: probes for molecular biology and autoimmune disease. Proc Natl Acad Sci U S A.

[pcbi-0030201-b055] Habets WJ, Hoet MH, De Jong BA, Van der Kemp A, Van Venrooij WJ (1989). Mapping of B cell epitopes on small nuclear ribonucleoproteins that react with human autoantibodies as well as with experimentally-induced mouse monoclonal antibodies. J Immunol.

[pcbi-0030201-b056] Reimer G, Pollard KM, Penning CA, Ochs RL, Lischwe MA (1987). Monoclonal autoantibody from a (New Zealand black × New Zealand white) F1 mouse and some human scleroderma sera target an Mr 34,000 nucleolar protein of the U3 RNP particle. Arthritis Rheum.

[pcbi-0030201-b057] Gama-Carvalho M, Carvalho MP, Kehlenbach A, Valcarcel J, Carmo-Fonseca M (2001). Nucleocytoplasmic shuttling of heterodimeric splicing factor U2AF. J Biol Chem.

[pcbi-0030201-b058] Press WH, Teukolsky SA, Vetterling WT, Flannery BP (1992). Random numbers. Numerical recipes in C: the art of scientific computing, 2nd edition.

[pcbi-0030201-b059] Matsumoto M, Nishimura T (1998). Mersenne Twister: a 623-dimensionally equidistributed uniform pseudo-random number generator. ACM Trans Model Comput Simul.

[pcbi-0030201-b060] Calapez A, Pereira HM, Calado A, Braga J, Rino J (2002). The intranuclear mobility of messenger RNA binding proteins is ATP dependent and temperature sensitive. J Cell Biol.

