# Supplementary figures and images for: A Stochastic View of Spliceosome Assembly and Recycling in the Nucleus
Source: PLoS Comput Biol. 2007 Oct 26;3(10):e201. doi: 10.1371/journal.pcbi.0030201 (PMC2041977; doi:10.1371/journal.pcbi.0030201)

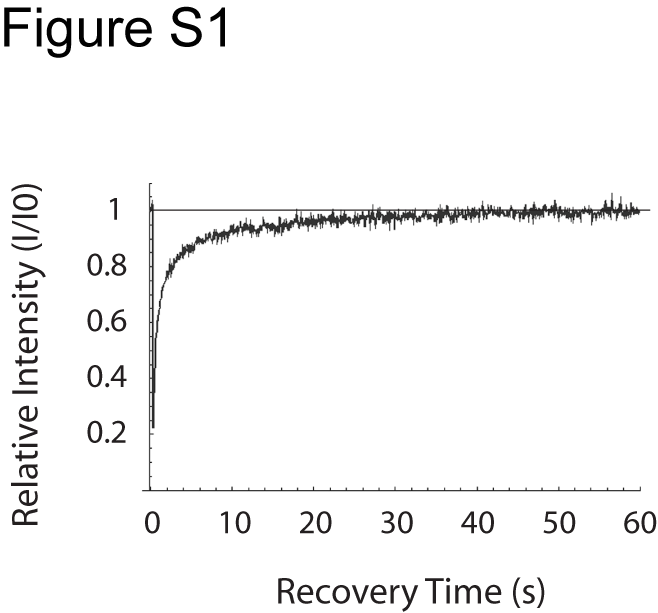

Supplement: Figure S1 — FRAP experiments were performed on HeLa cells expressing GFP-tagged U2AF65. The panel shows the FRAP recovery curve in nuclear speckles. This curve corresponds to a pool of three independent experiments, with ten different cells analyzed per experiment. (1.2 MB TIF) [file pcbi.0030201.sg001.tif]

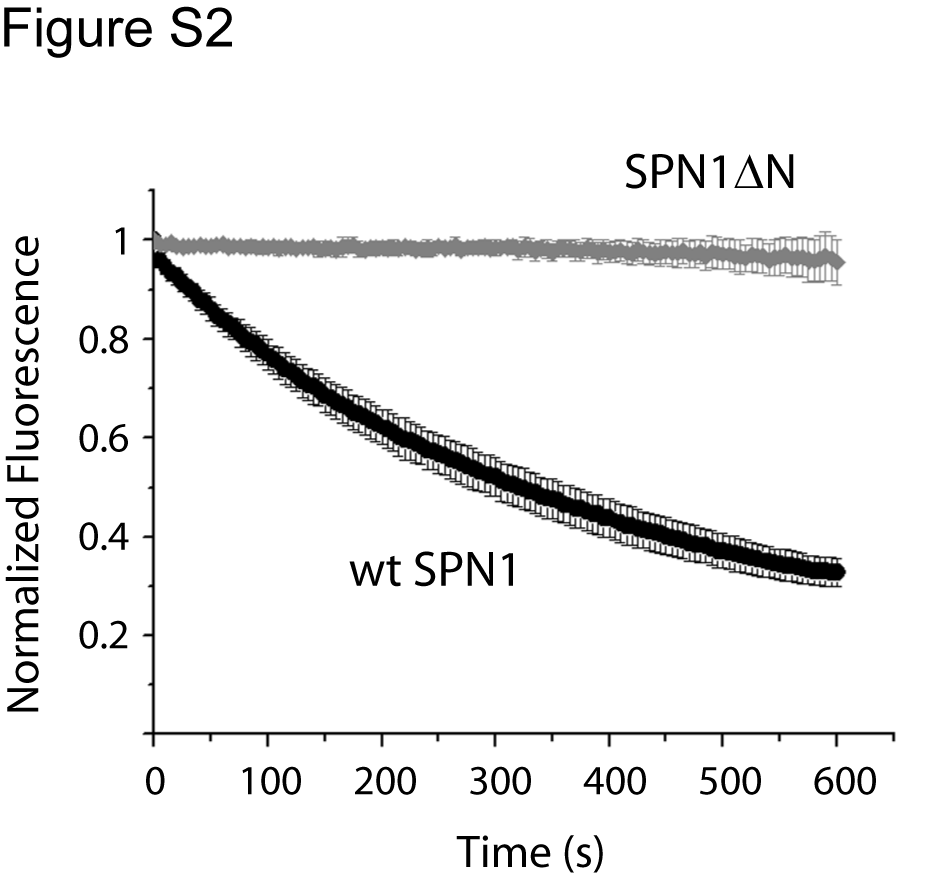

Supplement: Figure S2 — FLIP experiments were performed on HeLa cells expressing GFP-wt SPN1 or GFP-SPN1ΔN. The wild-type protein is predominantly detected in the cytoplasm. However, the fluorescence in the cytoplasm decays following repeated bleaching of the nucleus, indicating that the protein shuttles between the two compartments. In contrast, the intensity of GFP-SPN1ΔN fluorescence in the nucleus remains constant after repeated bleaching of the cytoplasm, indicating that this mutant protein is not shuttling. Each decay curve corresponds to six different cells analyzed. Fluorescence values were corrected for bleaching due to imaging, using ten different unbleached cells imaged in the same conditions. Error bars represent standard deviations. (2.4 MB TIF) [file pcbi.0030201.sg002.tif]

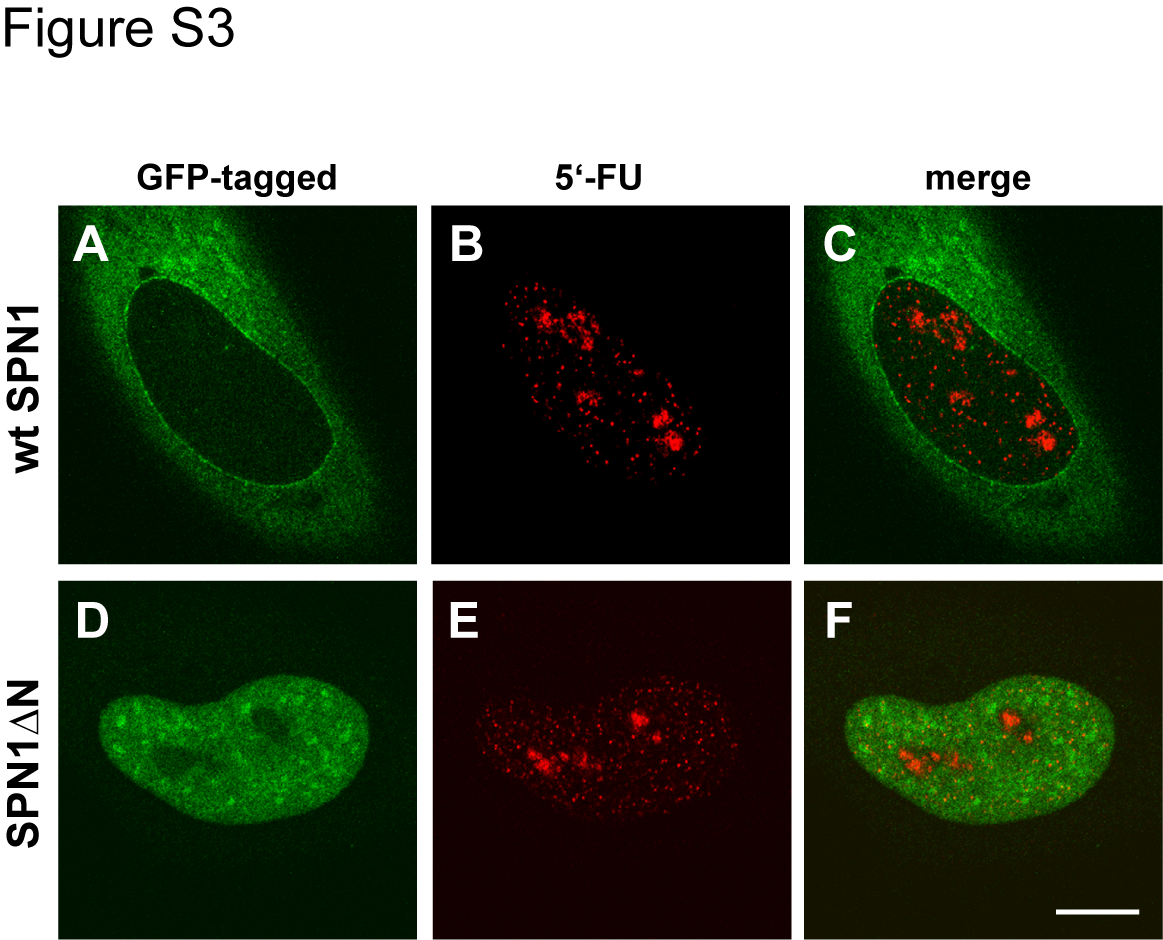

Supplement: Figure S3 — HeLa cells were transfected with either wtSPN1 (A–C) or the deletion mutant SPN1ΔN (D–F). Cells were incubated with 5′-fluoruridine (5'-FU) for 15 min. Living cells incorporate 5′-FU into nascent RNA, which is visualized with antibodies against halogenated nucleotides (red staining in (B,C,E,F)), showing that global transcription is active in both transfected cells. Bar indicates 10 μm. (3.2 MB TIF) [file pcbi.0030201.sg003.tif]

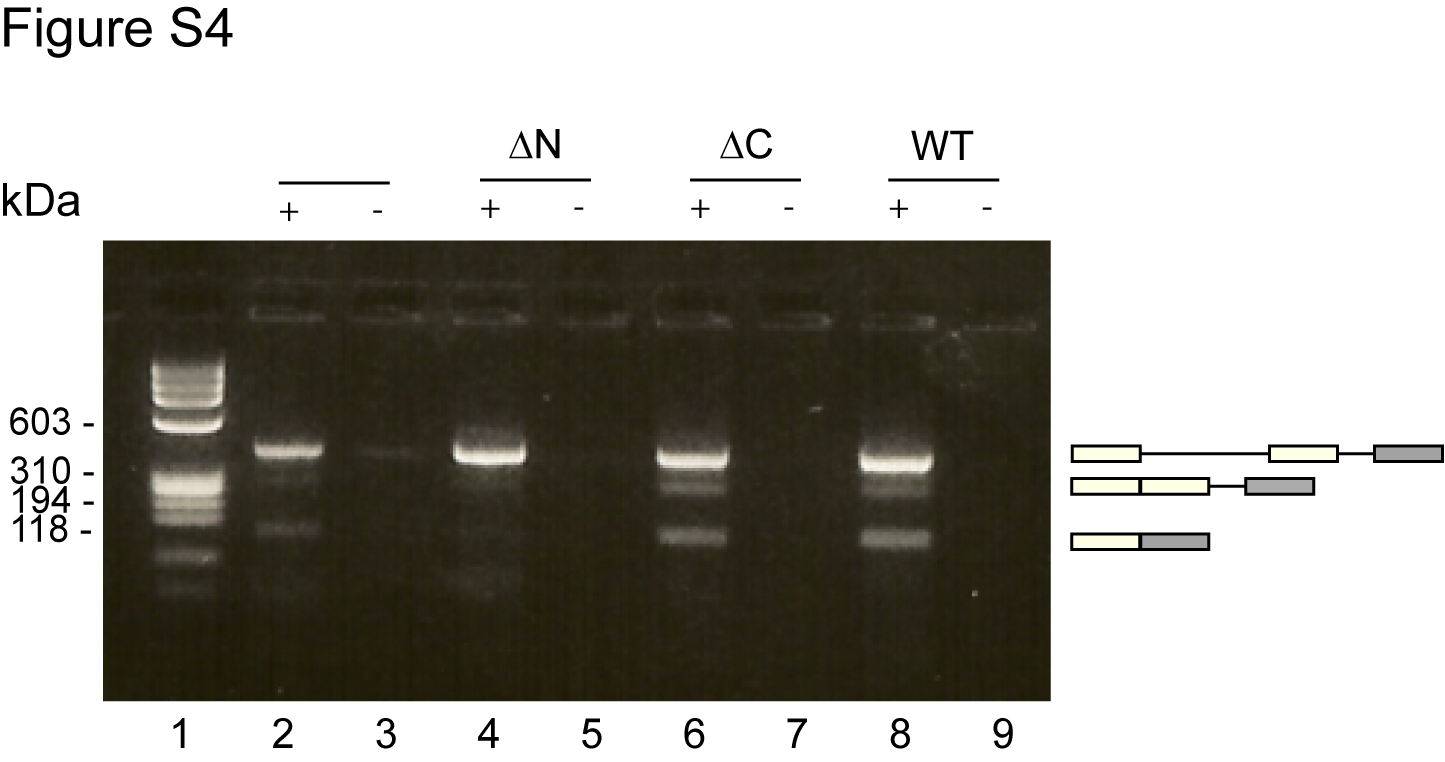

Supplement: Figure S4 — RT-PCR analysis of HeLa cells that were either transfected with a splicing reporter minigene (lanes 2 and 3) or cotransfected with the reporter minigene together with GFP-SPN1ΔN (lanes 4 and 5), GFP-SPN1ΔC (lanes 6 and 7), or GFP-wtSPN1 (lanes 8 and 9). Controls lacking reverse transcriptase indicate no contamination with plasmid DNA (lanes 3, 5, 7, and 9). The structure of each transcript is illustrated on the right. Molecular weight markers (kDa) are indicated on the left. (3.2 MB TIF) [file pcbi.0030201.sg004.tif]
